# Comparison of Two Solid-Phase Extraction (SPE) Methods for the Identification and Quantification of Porcine Retinal Protein Markers by LC-MS/MS

**DOI:** 10.3390/ijms19123847

**Published:** 2018-12-03

**Authors:** Carsten Schmelter, Sebastian Funke, Jana Treml, Anja Beschnitt, Natarajan Perumal, Caroline Manicam, Norbert Pfeiffer, Franz H. Grus

**Affiliations:** Department of Experimental and Translational Ophthalmology, University Medical Center, Johannes Gutenberg University, 55131 Mainz, Germany; cschmelter@eye-research.org (C.S.); sfunke@eye-research.org (S.F.); jana-treml@gmx.de (J.T.); a.beschnitt@gmx.net (A.B.); nperumal@eye-research.org (N.P.); caroline.manicam@unimedizin-mainz.de (C.M.); norbert.pfeiffer@unimedizin-mainz.de (N.P.)

**Keywords:** mass spectrometry, glaucoma animal model, biomarkers, sample clean-up, ZIPTIP^®^ C18 pipette tips, SOLAμTM HRP SPE spin plates

## Abstract

Proper sample preparation protocols represent a critical step for liquid chromatography-mass spectrometry (LC-MS)-based proteomic study designs and influence the speed, performance and automation of high-throughput data acquisition. The main objective of this study was to compare two commercial solid-phase extraction (SPE)-based sample preparation protocols (comprising SOLAµ^TM^ HRP SPE spin plates from Thermo Fisher Scientific and ZIPTIP^®^ C18 pipette tips from Merck Millipore) for analytical performance, reproducibility, and analysis speed. The house swine represents a promising animal model for studying human eye diseases including glaucoma and provides excellent requirements for the qualitative and quantitative MS-based comparison in terms of ocular proteomics. In total six technical replicates of two protein fractions [extracted with 0.1% dodecyl-ß-maltoside (DDM) or 1% trifluoroacetic acid (TFA)] of porcine retinal tissues were subjected to in-gel trypsin digestion and purified with both SPE-based workflows (*N* = 3) prior to LC-MS analysis. On average, 550 ± 70 proteins (1512 ± 199 peptides) and 305 ± 48 proteins (806 ± 144 peptides) were identified from DDM and TFA protein fractions, respectively, after ZIPTIP^®^ C18 purification, and SOLAµ^TM^ workflow resulted in the detection of 513 ± 55 proteins (1347 ± 180 peptides) and 300 ± 33 proteins (722 ± 87 peptides), respectively (FDR < 1%). Venn diagram analysis revealed an average overlap of 65 ± 2% (DDM fraction) and 69 ± 4% (TFA fraction) in protein identifications between both SPE-based methods. Quantitative analysis of 25 glaucoma-related protein markers also showed no significant differences (*P* > 0.05) regarding protein recovery between both SPE methods. However, only glaucoma-associated marker *MECP2* showed a significant (*P* = 0.02) higher abundance in ZIPTIP^®^-purified replicates in comparison to SOLAµ^TM^-treated study samples. Nevertheless, this result was not confirmed in the verification experiment using in-gel trypsin digestion of recombinant *MECP2* (*P* = 0.24). In conclusion, both SPE-based purification methods worked equally well in terms of analytical performance and reproducibility, whereas the analysis speed and the semi-automation of the SOLAµ^TM^ spin plates workflow is much more convenient in comparison to the ZIPTIP^®^ C18 method.

## 1. Introduction

Solid phase extraction (SPE) is a crucial technique in liquid chromatography-mass spectrometry (LC-MS)-based protein biomarker recovery, and therefore, the choice of appropriate SPE techniques must be planned carefully. Robustness, reproducibility, sensitivity, and economic parameters encompassing time and costs have to be addressed along with the selection of proper SPE protocols. There is an increasing number of SPE sorbent materials feasible for LC-MS-based proteomics in the current market, which compromises various formats; e.g., pipette tips, cartridges, discs, multi-well-plates, magnetic beads and sorbent materials, as reviewed elsewhere [1,2,3,4,5,6,7]. Since the implementation of SPE in LC-MS workflows should be cost- and time-effective while enhancing analysis speed, reproducibility, and minimizing processing errors, an important feature of a SPE technique is its ability for automation in compliance with a high-throughput proteomics methodology. Moreover, the SPE technique should be also compatible with the biological material of interest; for example, the retinal tissues in the present study. There is a growing demand to study the retina in the field of glaucoma proteomics, because it represents the primary ocular site that is affected in this neurodegenerative disorder, which is indicated by aberrant proteomic alterations [8]. To study these proteomic alterations LC-MS-based proteomic workflows represent the state-of-the-art strategy that provide sensitive and semi-quantitative information of the identified protein species [9,10,11]. This is particularly important for electrospray ionization (ESI) LC-MS-based proteomic workflows, in which the SPE platform is recommended for desalting, ionic detergent-removal (e.g., SDS) and peptide enrichment prior to LC-MS analysis and is mandatory for proper peptide ionization. Thus far, several different types of devices and sorbents have been developed and commercialized for proper sample clean-up procedures [12]. Our current LC-MS-based workflow for discovery proteomic approaches was successfully used for in-depth proteome characterization of retina samples from various species such as rat [13,14], pig [15] and human [16]. Recently, the LC-MS-based proteomic platform was also used for the quantitative proteomic analysis of a new established porcine retinal organ culture [17], which allows to monitor the expression levels of retinal proteins during experimental glaucomatous conditions. In the former retina proteomic studies, the ZIPTIP^®^ C18 pipette tip SPE was used as a standard operation protocol (SOP) for sample clean-up prior to LC-MS analysis. The purpose of the present work was to evaluate the suitability of a less laborious SPE method that can be implemented in the established LC-MS-based proteomic workflow and therefore, to compare the performance of SOLAµ^TM^ HRP SPE spin plates with the former ZIPTIP^®^ C18 pipette tips for the analysis of the porcine retinal proteome. Moreover, the main focus of the present study was on specific selected retinal protein markers, which were recently found to be associated with the pathophysiology of glaucoma [16] and represent interesting marker candidates for quantitative protein recovery. Both SPE techniques will be evaluated for specific protein/peptide recovery performance as well as for analysis speed of the porcine retinal proteome characterization and possibly considering an (semi-)automated integration of both SPE-based methods.

## 2. Results

### 2.1. 1-D SDS Page of Porcine Retinal Proteins

**Figure 1:**
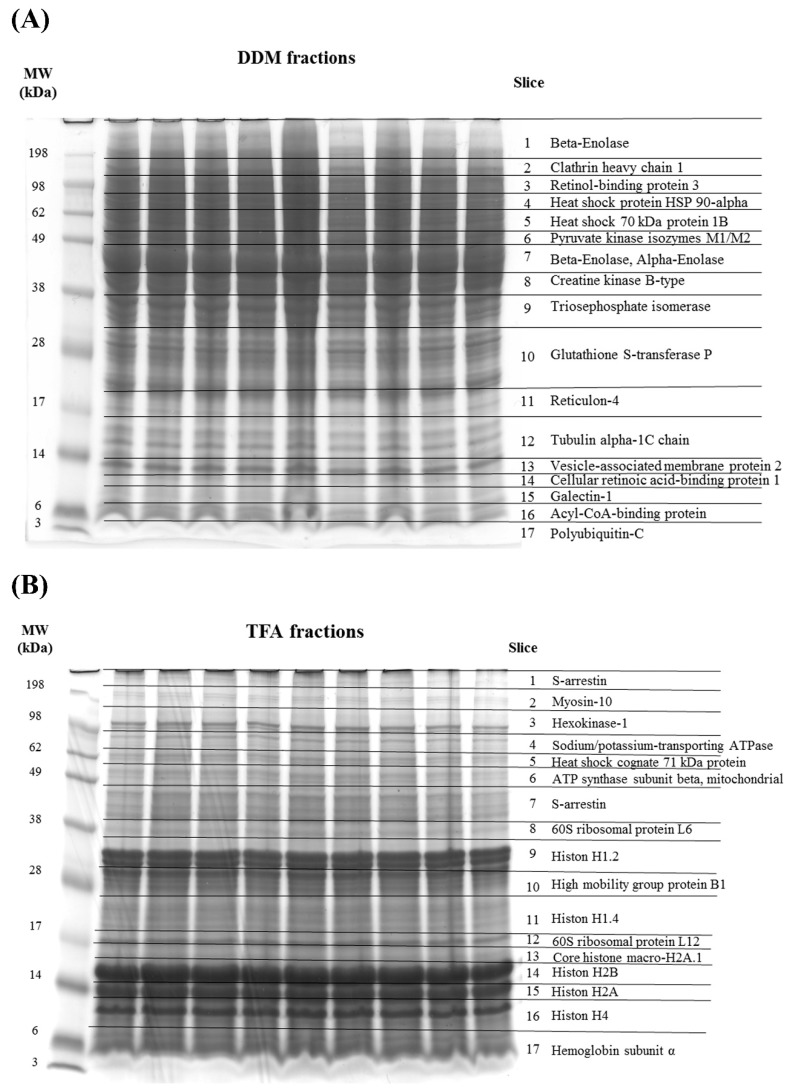
1-D SDS PAGE of porcine retinal proteins after extraction with 0.1% dodecyl-ß-maltoside (DDM) or 1% trifluoroacetic acid (TFA) buffer labeled with the most abundant proteins per spot. Each sample lane contains a total protein amount of 50 µg. (**A**) Protein migration pattern of the 0.1% DDM extract mostly containing cytoplasm-derived or membrane-associated proteins. (**B**) Protein migration pattern of the 1% TFA extract predominantly consists out of nucleus-derived proteins.

Both protein fractions (extracted with 0.1% DDM or 1% TFA) showed a high degree of congruency in the mass migration pattern with respect to the 1D SDS-PAGE (see Figure 1A,B). Each lane was subdivided into 17 slices labeled with the most abundant proteins per spot. Both protein fractions showed specific and reproducible protein pattern and particularly the DDM fraction contained predominantly cytoplasm-derived and membrane-associated proteins such as clathrin heavy chain 1 (≈150 kDa, highest protein score: 547, *CLTC*), retinol-binding protein 3 (≈98 kDa, highest protein score: 325, *RBP3*), glutathione S-transferase P (≈30–20 kDa, highest protein score: 1948, *GSTP1*) or reticulon-4 (≈17 kDa, highest protein score: 558, *RTN4*). In contrast, the TFA fraction exhibited mostly nucleus-derived proteins such as high mobility group protein B1 (≈28 kDa highest protein score: 818, *HMGB1*), different kinds of histones (e.g., Histone H1.4 ≈ 28–17 kDa, highest protein score: 3421, *HIST1H1E*), but also many mitochondrial (e.g., ATP synthase subunit beta, mitochondrial, ≈12 kDa, highest protein score: 1352, *ATP5B*) as well as ribosomal proteins (e.g., 60S ribosomal protein L12; ≈12 kDa, highest protein score: 589, *RPL12*).

### 2.2. Qualitative Comparison between Both SPE-Based Peptide Purification Methods

In order to evaluate qualitative differences regarding protein identifications, in total six technical replicates of two protein fractions (DDM and TFA) were purified with two different SPE-protocols (ZIPTIP^®^ and SOLAµ^TM^) prior to LC-MS/MS analysis (as described in detail in 2.4). On average the DDM fractions provided the identification of 550 ± 70 proteins (1512 ± 199 peptides) after ZIPTIP^®^ C18 purification protocol, whereas SOLAµ^TM^ purification workflow resulted in the identification of 513 ± 55 proteins (1347 ± 180 peptides) using high-confident filtering criteria (FDR < 1%). In contrast, the ZIPTIP^®^ C18 purification technique leads to the detection of 305 ± 48 proteins (806 ± 144 peptides) in the TFA fraction, whereas SOLAµ^TM^ purification protocol identified 300 ± 33 proteins (722 ± 87 peptides, see Supplementary Files S1 and S2). No significant differences (*P* > 0.05) regarding protein and peptide identifications were observed between both SPE-based purification methods using Student’s *t*-test.

**Figure 2:**
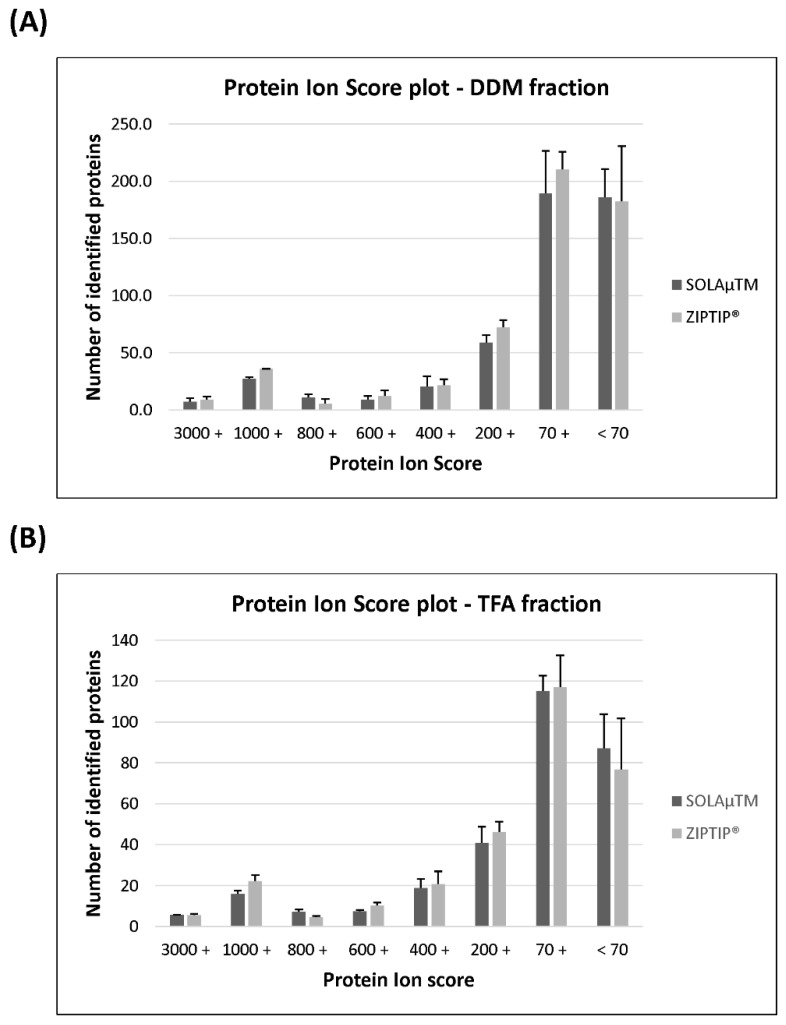
Distribution plot shows the classification of the identified proteins according to their specific protein ion scores. Proteins were clustered to the protein ion scores in the range of ≥3000, <3000 to ≥1000, <1000 to ≥800, <800 to ≥600, <400 to ≥200, <200 to ≥70 and <70. (**A**) Ion score distribution plot of the identified proteins in the DDM fraction after enrichment with to different solid phase extraction (SPE)-based purification methods (SOLAµ^TM^ and ZIPTIP^®^). (**B**) Distribution profile of the identified proteins in the TFA fraction according to their specific ion scores after enrichment with two different SPE methods.

**Figure 3:**
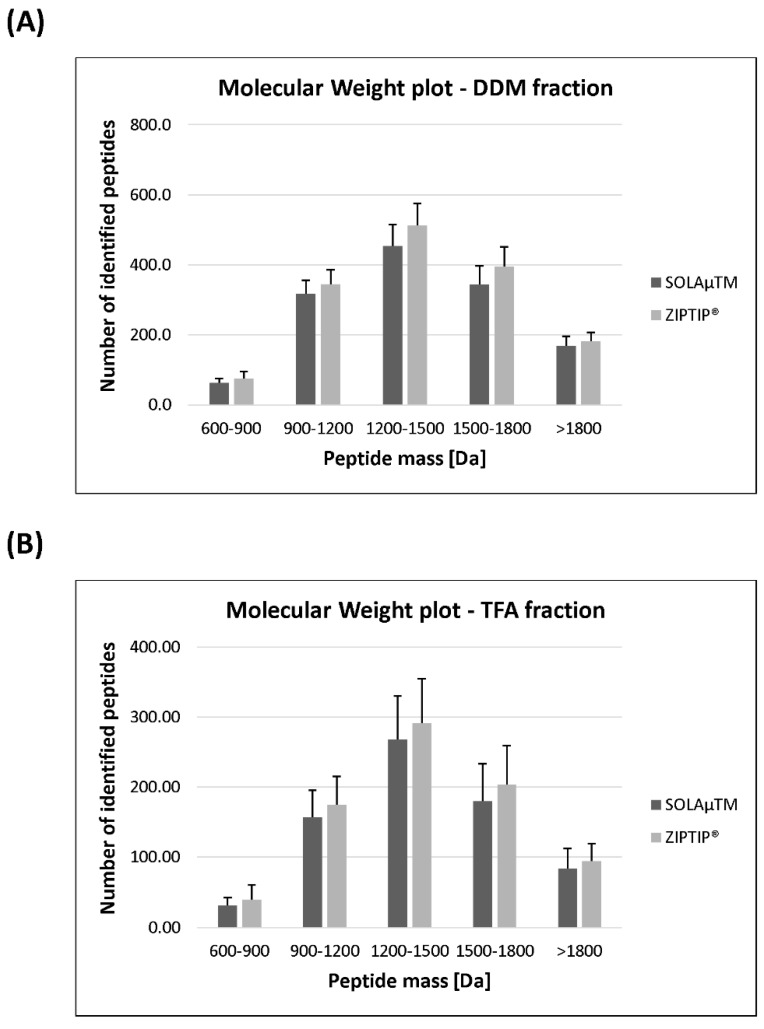
Distribution plot shows the classification of the identified peptides according to their characteristic molecular weight (MW). Peptides were clustered in the molecular mass range of ≥1800 Da, <1800 to ≥1500 Da, <1500 to ≥1200 Da, <1200 to ≥ 900 Da and <900 to ≥ 600 Da. (**A**) Bar plot shows the MW distribution of the identified peptides in the DDM fraction after enrichment with to different SPE-based purification methods (SOLAµ^TM^ and ZIPTIP^®^). (**B**) Distribution profiles of the MW of the identified peptides in the TFA fraction after enrichment with to different purification methods.

In the next step of the analysis, the identified proteins were classified according to their specific protein ion scores in order to evaluate if there are significant differences in the identification of group--specific proteins species (see Figure 2). On average, 7 ± 3 proteins of the SOLAµ^TM^-purified DDM fraction showed a protein score of >3000. Up to 318 ± 70 proteins indicated a protein score of <3000 >70 and 186 ± 25 proteins were detected with a protein score of <70. On the other hand, the ZIPTIP^®^ C18 procedure resulted in the detection 9 ± 3 proteins with a protein score of >3000 in the DDM fraction. On average 359 ± 78 proteins showed a protein score of <3000 >70 and up to 183 ± 48 proteins were identified with a protein score of <70. Nevertheless, no significant group-specific differences (Mann-Whitney U-test, *P* > 0.05) in terms of the protein ion score were observed between both SPE methods. The proteins of the TFA fraction showed a similar distribution pattern with respect to the protein ion score, but also no significant changes (Mann–Whitney U-test, *P* > 0.05) were observed between both methods. Furthermore, we also clustered the identified peptides according to their characteristic molecular weights (MW), in order to analyze if there are any significant differences in the detection of group-specific peptide sequences between both purification protocols (see Figure 3). On average both SPE workflows resulted in comparable peptide identification rates (SOLAµ^TM^: 1115 ± 149 peptides; ZIPTIP^®^: 1254 ± 156 peptides) within the mass range of 900 to 1800 Da in the DDM fraction. With both SPE methods the number of identified peptides (SOLAµ^TM^: 232 ± 31 peptides; ZIPTIP^®^: 258 ± 45 peptides) decreased at the edges of the mass range between 600-900 Da and >1800 Da. Nevertheless, none of the groups showed significant differences (Student’s *t*-test, *P* > 0.05) between both purification methods in the DDM fraction. In addition, the MW-classified peptides of the TFA fraction showed a similar distribution pattern, but also no significant differences (Student’s *t*-test, *P* > 0.05) were found between SOLAµ^TM^ - and ZIPTIP^®^-purified study samples.

**Figure 4:**
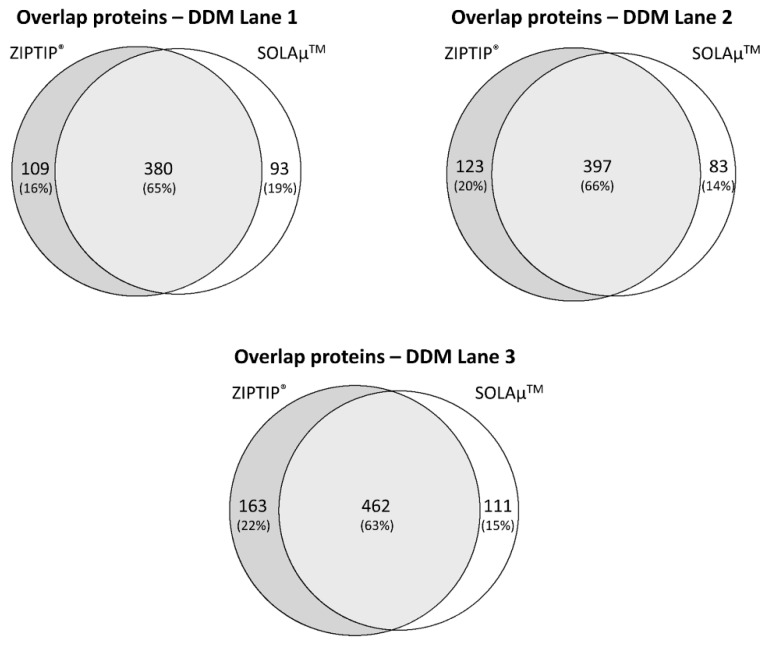
Venn diagram showing the overlap (percentage distribution) of identified proteins in the DDM fraction between six technical replicates. Three technical replicates were either purified by ZIPTIP^®^ pipette tips or SOLAµ^TM^ microtiter plates. On average 65 ± 2% of all identified proteins were detected with both SPE-based purification methods.

**Figure 5:**
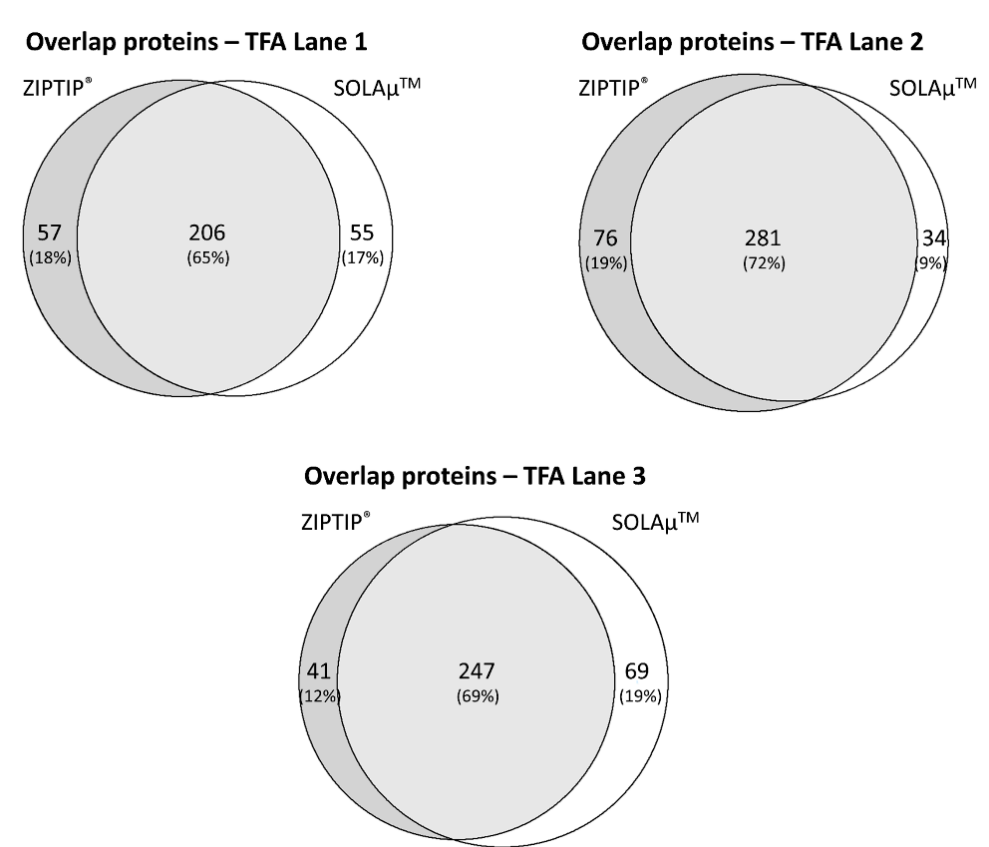
Venn diagram showing the overlap (percentage distribution) of identified proteins in the TFA fraction between six technical replicates. Three technical replicates were either purified by ZIPTIP^®^ pipette tips or SOLAµ^TM^ microtiter plates. On average 69 ± 4% of all identified proteins were detected with both SPE-based purification methods.

**Figure 6:**
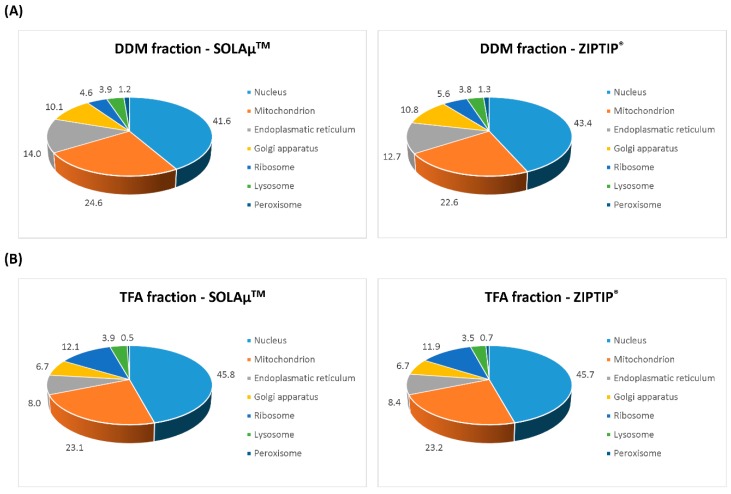
Gene ontology (GO) annotation analysis of all identified porcine retinal proteins. Proteins were clustered according to their cellular compartments such as nucleus, mitochondrion, endoplasmatic reticulum, golgi apparatus, ribosome, lysosome or peroxisome. DDM (**A**) as well as TFA fraction (**B**) did not show any conspicuous differences regarding the cellular localization of the identified proteins between both SPE-based purification methods (SOLAµ^TM^ and ZIPTIP^®^).

Venn diagram analysis revealed that on average 65 ± 2% of all identified proteins were detected with both SPE-based purification methods (SOLAµ^TM^ and ZIPTIP^®^) in the DDM fraction (Figure 4). Also, the TFA fraction provided an average overlap of 69 ± 4% of all identified proteins between both purification methods (Figure 5). Even on the peptide level both methods resulted in an overlap of 60 ± 1% in the DDM fraction and an overlap of 62 ± 5% in the TFA fraction (Supplementary Figures S1 and S2). Furthermore, around 98% of all identified proteins in both protein fractions (DDM and TFA) could be annotated to cellular compartments according to the gene ontology (GO) analysis. Additionally, the identified proteins from the DDM and TFA fractions did not show any conspicuous differences regarding the cellular localization between both SPE-based purification methods (Figure 6). 

### 2.3. Quantitative Analysis between Both SPE-Based Peptide Purification Methods

**Figure 7:**
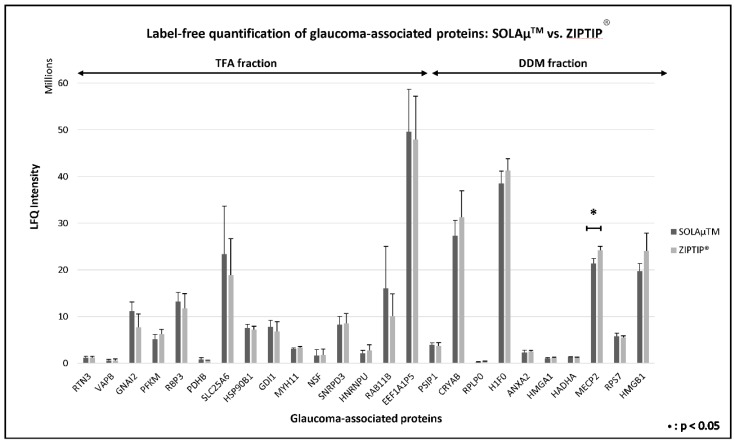
Bar plot shows the label-free quantification results of 25 glaucoma-associated biomarker candidates after enrichment with two different SPE purification methods (SOLAµ^TM^ and ZIPTIP^®^). Marker candidates on the left were identified in the DDM fraction, whereas proteins on the right were detected in the TFA fraction (FDR < 1%). Only protein *MECP2* showed a significant (*p* < 0.05) higher abundance in ZIPTIP^®^-purified replicates in comparison to SOLAµ^TM^-purified study samples.

The aim of the second part of this study was to identify if there are significant differences in the label-free quantification (LFQ) of proteins between both SOLAµ^TM^ and ZIPTIP^®^ C18 purification methods. For the quantitative analysis, several glaucoma-associated retinal biomarker candidates were selected, which showed different expression levels (*P* < 0.1) in retinal tissues of glaucoma patients in contrast to healthy controls [16]. Up to 15 of these glaucoma-associated biomarker candidates were identified in the DDM fraction, whereas the TFA fraction contained up to 10 retinal biomarker candidates (Figure 7 and Supplementary Table S1). The three most abundant marker candidates of the DDM fraction were elongation factor 1-alpha 1 (*EEF1A1*), ADP/ATP translocase 3 (*SLC25A6*) and ras-related protein Rab-11B (*RAB11B*). In contrast, the TFA fraction provided the three most abundant biomarker candidates histone H1.0 (*H1F0*), α-crystallin B chain (*CRYAB*) and methyl-CpG-binding protein 2 (*MECP2*). Approximately 96% of the identified biomarker candidates did not show any significant differences (Student’s t-test, *P* > 0.05) in the LFQ analysis between both SPE purification methods. Only protein marker *MECP2* showed a significant higher abundance (Student’s *t*-test, *P* = 0.02) in ZIPTIP^®^-purified replicates in contrast to SOLAµ^TM^-purified study samples. Verification experiment with tryptic in-gel digestion of recombinant protein *MECP2* (see Supplementary Figure S3) resulted in a slightly higher abundance in the ZIPTIP^®^-purified study samples (LFQ intensity=11.5 × 10^7^ ± 2.7 × 10^7^, *N* = 9) compared to SOLAµ^TM^-purified replicates (LFQ intensity=10.1 × 10^7^ ± 2.2 × 10^7^, *N* = 9), however no significant difference (Student’s *t*-test, *P* = 0.24) was found.

## 3. Discussion

The LC-MS-based proteomic analysis of the porcine retinal tissue samples provided a sensitive view into the complex porcine retina proteome and is in confidence with recent protein catalogues of the pig retina [15,18,19,20]. The present study contributes to a better characterization of the pig eye proteome and promotes the house swine (*Sus scrofa*) as a suitable candidate for ocular disease model systems [21,22,23,24]. However, the “core proteome” of the pig retina was congruently characterized with both SPE methods via ZIPTIP^®^ C18 pipette tips or SOLAµ^TM^ HRP spin plates (see Supplementary Files S1 and S2), facilitating the LC-MS-based detection of high abundant proteins such as clathrin heavy chain 1 (*CLTC*), pyruvate kinase isozymes M1/M2 (*PKM*), creatine kinase B-type (*CKB*), glyceraldehyde-3-phosphate dehydrogenase (*GAPDH*), rhodopsin (*RHO*), various tubulin chains as well as 14-3-3 proteins. Moreover, subcellular compartments were comparably distinguished with both purification techniques, highlighting nucleus and mitochondria as the main annotated organelles (see Figure 6). Also, both systems worked equally well concerning the identification of retinal proteins from two different protein fractions: DDM- and TFA-containing solvents provided a high overlap regarding protein and peptides identifications between both purification techniques (see Figure 4 and Figure 5; see Supplementary Figures S1 and S2). Tabb et al. (2010) [25] observed that peptide lists from several technical replicates overlapped from 35 to 60% and is in accordance with the results of the present study. The loss of repeatability in such LC-MS-based proteomic study designs is caused due to the complexity of the sample, ion suppression effects, precursor fragmentation efficiency or several others factors as described in detail elsewhere [25,26,27,28]. Nevertheless, both SPE protocols also showed a certain degree of contrary indicated by ZIPTIP^®^ C18- and SOLAµ^TM^-HRP exclusive protein species (e.g., ZIPTIP^®^: *VAMP2* and SOLAµ^TM^: *HSP90AB1*, see Supplementary File S1). In accordance, the ZIPTIP^®^ C18 method showed a slightly increased detection rate of proteins not included in the “core proteome” accompanied by slight tendencies of higher peptide ion scores and a higher sensitivity over the inspected mass range (see Figure 3 and Figure 4). However, since none of these effects was supported by statistical significance (*P* > 0.05), an almost equal analytical performance and reproducibility of both SPE workflows can be concluded.

In terms of quantitative recovery of the glaucoma-associated retinal target proteins, there were also no distinctive statistical differences (*P* > 0.05) between both SPE methods (see Figure 7). However, for some focused retinal proteins, e.g., ADP/ATP translocase 3 (*SLC25A6*), guanine nucleotide-binding protein G(i) subunit α-2 (*GNAI2*) or Ras-related protein Rab-11B (*Rab11B*), the SOLAµ^TM^ SPE method showed tendencies of quantitatively higher protein recovery, whereas for other target proteins, e.g., high mobility group protein B1 (*HMGB1*) or α-crystallin B chain (*CRYAB*), the ZIPTIP^®^ C18 technique achieved a slightly trend to quantitatively higher protein abundances. Nevertheless, since all of these findings were not supported by statistical significance (*P* > 0.05) an approximately comparable protein/peptide recovery performance of the two tested SPE methods can be assumed. Exceptions are the results regarding *MECP2* extraction, since *MECP2* was significantly (*P* = 0.02) recovered in higher quantities from retinal samples purified by the use of ZIPTIP^®^ C18 pipette tips. However, this effect could not be reproduced by verification experiment (*P* = 0.24) considering the peptide enrichment of tryptic, recombinant MECP2 with both SPE devices. The different performance in *MECP2* extraction may emphasize the special retention behavior of this protein. As a member of intrinsically disordered proteins (IDPs), *MECP2* lacks higher structural organization [29,30,31] providing high plasticity for molecular interactions [32], which is reflected by its amino acid sequence. This may have also influenced the retention behavior of *MECP2-*derived peptides on SPE sorbents; e.g., on HRP representing a polymeric sorbent with polar and non-polar retention properties [33].

The SOLAµ^TM^ HRP spin plate workflow is clearly a superior method compared to the manual ZIPTIP^®^ C18 protocol with respect to the effort, costs, and analysis time factors. Although the ZIPTIP^®^ C18 method can be automated on robotic stations [34], the speed and robustness of such a fully automated SPE pipette tip system is hampered by the lack of resistance towards errors such as trapped air-bubbles in the resin tips or pipette tips blocked by sample debris. This error-prone procedure can be avoided by using the SOLAµ^TM^ HRP spin plates due to the macro-porous structure of the solid phase material, which provides continuous solvent flow-through without any sample loss [35]. Furthermore, the time factor is crucial in fully automated SPE pipette tip platforms in regard to solvent diffusion, alterations in solvent composition, robot movement distances and robot working cycles. In accordance, the constant need for trouble shooting in such SPE pipette tip automation systems is an important limitation compared to the centrifugal-based SPE spin plates. Indeed, the execution of the SOLAµ^TM^ HRP spin plate workflow still requires a certain degree of operator handling and represents only a semi-automatic platform, nevertheless, it provides a higher degree of standardization and reproducibility than the manual ZIPTIP^®^ C18 pipette tip workflow. However, due to the semi-automatic system properties and the consequent compatibility with LC-MS-based high-throughput screening strategies, centrifugal SPE spin plates have recently been “expected to become the mainstream method of sample processing in the future” [36] and were successfully used in various proteomic study designs focusing on human cerebrospinal fluid [37], human serum [38], human lung tissues [39] and HeLa cells [40]. 

In conclusion, the SOLAµ^TM^ HRP spin plate approach represents an attractive alternative to the manual ZIPTIP^®^ C18 pipette tip workflow and is particularly recommended for high-throughput discovery proteomic platforms to enhance cost-, labor- and time-effectiveness purposes. Nevertheless, considering the sensitive performance of the manually applied ZIPTIP^®^ C18 tips, a targeted use of these pipette tips for the analysis of specific marker proteins such as *SLC25A6*, *GNAI2*, *Rab11B* or *MECP2* should be considered. In addition, the development of a mixed-mode spin plate, benefiting from the properties of both stationary materials (HRP and C18), could be a future innovation for improved recovery of retinal proteins. Beyond that, fine-tuning adjustments of the SOLAµ^TM^ HRP spin plate workflow such as centrifugal speed, time, temperature, elution volume, elution buffer and number of repeat cycles could significantly improve the sensitivity and accuracy of the current proteomic measurements.

## 4. Materials and Methods

### 4.1. Sample Preparation and Protein Extraction Protocols

Retinal tissues were prepared from freshly enucleated eye bulbs (*N* = 45) from house swine *Sus scrofa domestica* Linnaeus, 1758 individuals (sacrificed at 3–6 month, female:male = 3:2) provided by local slaughterhouses (Landmetzgerei Harth, Stadecken-Elsheim, Germany; Metzgerei Köppel, Mainz, Germany). Eye bulbs were equatorially opened with a scalpel to remove lens, vitreous body, iris and ciliary body. Retinal tissues were carefully removed from the pigment epithelium with a phosphate-buffered saline (PBS)-coated paintbrush. After this, the whole retina was cut off from the optic nerve head and stored at −80 °C until further protein extraction protocols. For the first protein extraction protocol 30 isolated retina tissues were pooled and homogenized with an Ultra-Turrax T25 sonicator (Janke & Kunkel IKA Labortechnik, Staufen im Breisgau, Germany) as described in detail in an earlier study [15]. After homogenization, 0.1% dodecyl-ß-maltoside (DDM) was added to the aliquots and sonicated for 10 min on ice. Then, the samples were gently mixed and incubated for 30 min at RT followed by centrifugation at 10,000× *g* for 12 min at 4 °C. The supernatant was collected and stored at −20 °C. For the second protein extraction protocol 15 isolated retina tissues were pooled and subjected to further homogenization using a Precellys^®^ 24 homogenizer (VWR International GmbH, Darmstadt, Germany) combined with a 1.4/2.8 mm Precellys^®^ Ceramic kit (VWR International GmbH, Darmstadt, Germany). Prior to homogenization, frozen retina tissues were added to the tubes containing the 1.4/2.8 mm ceramic balls and filled with 1.5 mL PBS. Retina samples were homogenized three times for 45 s at 5000 rpm and centrifuged afterwards at 10,000× *g* for 12 min at 4 °C. The supernatant containing mostly cytoplasm-derived proteins was stored at −20 °C and the remaining pellet was resuspended in 500 µL of 1% trifluoroacetic acid (TFA). Funke et al. (2016) [15] have previously shown that 1% TFA is a suitable buffer for the extraction of many nucleus-derived retinal proteins. Retina samples were homogenized three times for 45 s at 5000 rpm followed by centrifugation at 10,000× *g* for 12 min at 4 °C. The supernatant containing mostly nucleus-derived proteins was stored at −20 °C. Protein measurements of both protein fractions were performed using BCA protein assay kit (Thermo Fisher Scientific, Rockford, IL, USA) according to manufacturer’s instructions and measured three times with a Multiscan Ascent photometer (Thermo Fisher Scientific, Rockford, IL, USA) at a wavelength of 570 nm. Human Recombinant protein *MECP2* (Methyl-CpG-binding protein 2, cat. no. 14-1067) was purchased from Merck Millipore (Billerica, MA, USA) and used for the validation experiment.

### 4.2. 1-D SDS Page

Both protein fractions (50 µg per lane) were separated on 10-well NuPAGE 12% Bis-Tris minigels (Thermo Fisher Scientific, Rockford, IL, USA) under reducing conditions. Gels were incorporated in the XCell SureLock™ Mini-Cell Electrophoresis System (Invitrogen, Carlsbad, CA, USA) and prepared with NuPAGE™ MOPS SDS Running Buffer 20× (Thermo Fisher Scientific, Rockford, IL, USA) according to the supplier’s protocol. In addition, 10 µL of the SeabluePlus 2 Pre-Stained Protein Standard (Thermo Fisher Scientific, Rockford, IL, USA) was used as molecular weight reference and separated at 150 V for 1.5 h at 4 °C. After separation, gels were fixed and stained using Novex Colloidal Blue Staining Kit (Thermo Fisher Scientific, Rockford, IL, USA) according to the manufacturer’s instructions. Gels were destained for at least 16 h and scanned using an Epson Perfection V600 Photo Scanner (Seiko Epson Corporation, Suma, Nagano, Japan) at 700 dpi. Protein migration patterns were manually inspected and subjected to further in-gel trypsin digestion. 

### 4.3. In-gel Trypsin Digestion

In total six lanes of both protein fractions were subjected to further in-gel trypsin digestion according to a modified protocol from a previous study [41]. At first, each protein lane was subdivided into 17 slices according to their characteristic protein migration profile and cut into small pieces. The gel pieces were destained with 100 mM ammonium bicarbonate (ABC) in 50% acetonitrile (ACN) and dehydrated with pure ACN before reduction and alkylation processes. Then, the samples were evaporated in the SpeedVac (Eppendorf, Darmstadt, Germany) for 10 min at 30 °C to dryness. Afterwards the gel pieces were resolved with 10 mM dithiothreitol (DTT) in 100 mM ABC and incubated for 30 min at 56 °C followed by incubation with 55 mM iodoacetamide (IAA) in 100 mM ABC for 30 min at RT in the dark. Then the samples were dehydrated one more time with pure ACN and dried for 10 min in the SpeedVac at 30 °C to dryness. The reduced and alkylated proteins were further digested with 10 µg/mL sequencing grade trypsin (Promega, Madison, WI, USA) in 10 mM ABC 10% ACN and incubated overnight for at least 16 h at 37 °C. On the next day, the supernatant was collected and the tryptic peptides were extracted with 10% formic acid (FA) 70% ACN for 30 min at 350 rpm. Both supernatant fractions were pooled and evaporated in the SpeedVac at 30 °C to dryness.

### 4.4. SPE-Based Peptide Purification

The extracted peptides were dissolved in 20 µL 0.1% TFA. In order to reduce technical variability during execution of the sample preparation protocols, the slices (*N* = 17) of two lanes (DDM or TFA protein fractions) were pooled to a total volume of 40 µL 0.1% TFA. Then, the peptide pools were subsequently split into two equal amounts (each 20 µL) and subjected for further SPE purification via SOLAµ™ SPE HRP plates (Thermo Fisher Scientific, Rockford, IL, USA) or via C18 SPE pipette tips (Merck Millipore, Billerica, MA, USA). In total three technical replicates of each protein fraction (DDM or TFA) were purified with both SPE methods. Purification via ZIPTIP^®^ C18 SPE pipette tips represents the SOP in our lab and was performed according to previous publications [9,10,11]. In brief, the ZIPTIP^®^ C18 SPE pipette tips were conditioned and equilibrated by pipetting 10 µL ACN three times followed by 10 µL 0.1% TFA three times. Then the sample was loaded on the stationary C18 phase by aspirate and dispense 20 times, washed three times with 0.1% TFA and finally eluted two times in 10 µL 50% ACN 0.1% TFA. This procedure was repeated, the pooled eluate fractions (40 µL) were evaporated in the SpeedVac at 30 °C to dryness and stored at −20 °C. The SPE-based peptide purification via SOLAµ™ SPE HRP plates was performed according to the manufacturer’s instructions. Activation and elution of the SOLAµ^TM^ SPE membranes was performed with 100 µL methanol (MeOH), whereas the washing step was performed with 100 µL 5% MeOH. After each step of the sample preparation protocol the SOLAµ^TM^ SPE plate was centrifuged at 4000× *g* for 3 min and the flow-through/eluate fractions were collected in 96-well microtiter microplates (Costar Corning Incorporated, Corning, NY, USA). Eluates were transferred into new reaction tubes, evaporated in the SpeedVac at 30 °C to dryness and stored at −20 °C prior to further LC-MS/MS analysis.

### 4.5. LC-MS/MS Analysis

LC-MS measurements were performed by a Rheos Allegro pump (Thermo Fisher Scientific, Rockford, IL, USA) downscaled to a capillary HPLC system (flow rate: 6.7 ± 0.3 µL/min) online coupled to a hybrid linear ion trap - Orbitrap MS (LTQ Orbitrap XL; Thermo Fisher Scientific, Rockford, IL, USA). Purified tryptic peptides were resolved in 10 µL 0.1 TFA and 6 µL were injected into a BioBasic^®^ C18 column system (30 × 0.5 mm pre-column + 150 × 0.5 mm analytical column; Thermo Fisher Scientific, Rockford, IL, USA). Solvent A consists of 1.94% ACN, 0.06% MeOH, and 0.05% FA in water and solvent B consists of 95% ACN, 3% MeOH and 0.05% FA in water. Peptides were eluted within 50 min using following gradient program: 15–20% B (0–2 min), 20–60% B (2–35 min), 60–100% B (35–40 min), 100–0% B (40–45 min), and 0% B (45–50 min). The LTQ Orbitrap operated in positive ionization mode and data-dependent acquisition (DDA) mode: High-resolution survey full scan (from *m/z* 300 to 2000) was performed in the Orbitrap with a resolution of 30.000 at 400 *m/z* and the target automatic gain control was set to 1 × 10^6^ ions. For internal calibration the lock mass was set to 445.120025 *m/z* (polydimethylcyclosiloxane). Dynamic exclusion mode was enabled with the following settings: repeat count = 1, repeat duration = 30 s, exclusion list size = 100, exclusion duration = 90 s and exclusion mass width= ± 20 ppm. Based on the high-resolution MS scan the five most intense precursor ions were selected for further collision-induced dissociation (CID) fragmentation in the ion trap employing normalized collision energy of 35%. Manual inspection of the total ion current (TIC) chromatogram was performed using Qual Browser v. 2.0.7 SP1 (Thermo Fisher Scientific, Rockford, IL, USA). LC-MS raw data were uploaded to the ProteomeXchange Consortium via the PRIDE [42] partner repository with the dataset identifier PXD011755.

### 4.6. Peptide Identification and Quantification

For protein identification acquired LC-MS profiles were analyzed with software package Proteome Discoverer (Version 1.1; Thermo Fisher Scientific, Rockford, IL, USA) using the mascot search engine (version 2.2.07) to obtain peptide scoring information. Tandem MS spectra were searched against SwissProt database (SwissProt_150301) with a combination of *Homo sapiens* and *Sus scrofa* as taxonomies with following settings: peptide mass tolerance of ± 30 ppm in the range of 150–2000 Da, fragment mass tolerance of ± 0.5 Da, tryptic cleavage, a maximum of one missed cleavages, carbamidomethylation as fixed modification, acetylation (Protein N-terminal) and oxidation as variable modification. Output data were filtered considering a false discovery rate (FDR) < 1%. Given the fact that there is a limited access to proper public proteomic databases of the house swine (*Sus scrofa*) [43,44], we included the species-related proteomic database of *Homo sapiens* for protein search in order to maximize the protein identification results. Label-free quantification (LFQ) of the proteins was performed with MaxQuant computational proteomics platform version 1.5.2.8 (Max Planck Institute of Biochemistry, Martinsried, Germany). Tandem MS spectra were searched against a user-defined database, containing glaucoma-associated proteins (SwissProt_170531), with the previously described database search settings. In addition, MaxQuant specific feature “match between run” was enabled and proteins were identified considering FDR < 1%. Glaucoma-associated human protein database contains a selection of retinal protein sequences which showed at least a tendency (*P* < 0.1) to be differentially expressed between glaucoma patients and healthy controls according to a recent publication [16]. 

### 4.7. Data Analysis

Graphical presentation and *t*-test statistics for the qualitative analysis of the MS output data was performed with software package Statistica version 13 (Statsoft, Tulsa, OK, USA). Normal distribution of the data-sets was verified by the Shapiro–Wilk test. Student’s *t*-test was applied for parametric data and Mann–Whitney U-test for non-parametric data. Venn diagram analysis was performed with statistics program R version 3.2.0 with VennDiagram package version 1.6.17 (Available online: https://www.r-project.org/). Combined protein lists from the SOLAµ^TM^- and ZIPTIP^®^-purified protein fractions were subjected to gene ontology (GO) analysis using software program Cytoscape version 2.8.3 with BINGO 2.44 plugin ((Available online: www.cytoscape.org). Thereby, the GO category “cellular component” was screened for potential annotations. Statistical analysis of the MaxQuant generated output data was performed using software package Perseus version 1.5.5.0 (Max Planck Institute of Biochemistry, Martinsried, Germany). At first, LFQ intensities of the detected proteins were log_2_ transformed for further analysis [45]. Prior to statistical analysis the output data were filtered for contaminants, reversed hits, “only identified by site” and for a minimum number of three valid values in at least in one group. Finally, two-sided *t*-test statistics with *P* values <0.05 was applied in order to identify significant level changes in protein abundances between both SPE-based purification methods (SOLAµ^TM^ and ZIPTIP^®^).

## Supplementary Materials

Supplementary materials can be found at http://www.mdpi.com/1422-0067/19/12/3847/s1. A list of all identified proteins and peptides is provided in Supplementary Files S1 and S2. The mass spectrometry proteomics data have been deposited to the ProteomeXchange Consortium via the PRIDE [42] partner repository with the dataset identifier PXD011755.
