# Supplementary material for: Comparison of Two Solid-Phase Extraction (SPE) Methods for the Identification and Quantification of Porcine Retinal Protein Markers by LC-MS/MS
Source: Int J Mol Sci. 2018 Dec 3;19(12):3847. doi: 10.3390/ijms19123847 (PMC6321002; doi:10.3390/ijms19123847)
Supplement: Supplementary file 1 [file ijms-19-03847-s001.zip › supplementary files/Supplementary figures.docx]

Supplementary figures


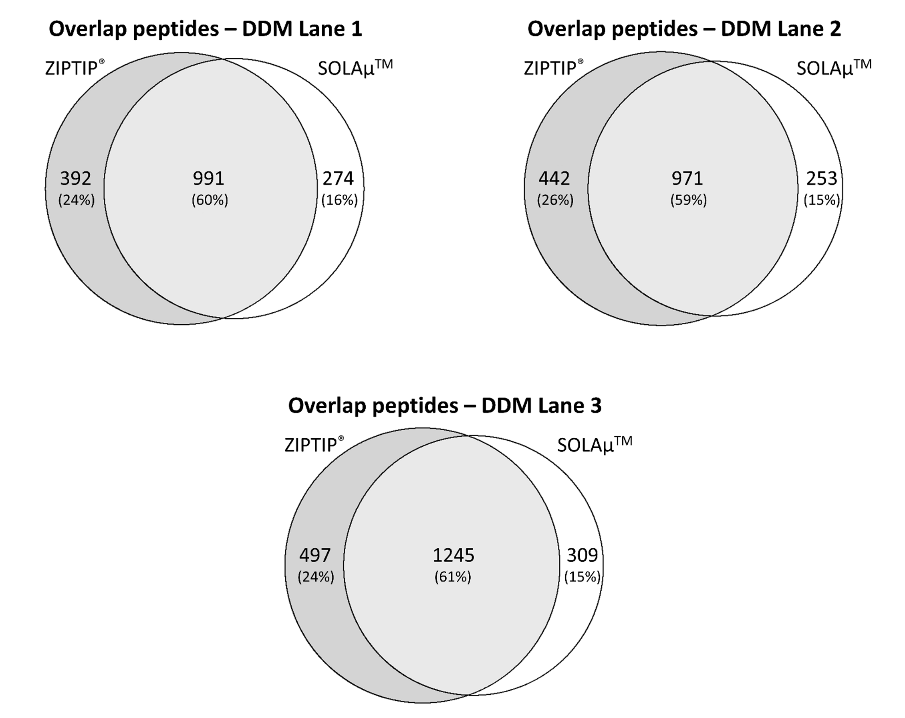


**Supplementary Fig. 1:** Venn diagram showing the overlap (percentage distribution) of identified peptides in the DDM fraction between six technical replicates. Three technical replicates were either purified by ZIPTIP^®^ pipette tips or SOLAµ^TM^ microtiter plates. On average 60±1 % of all identified peptides were detected with both SPE-based purification methods.


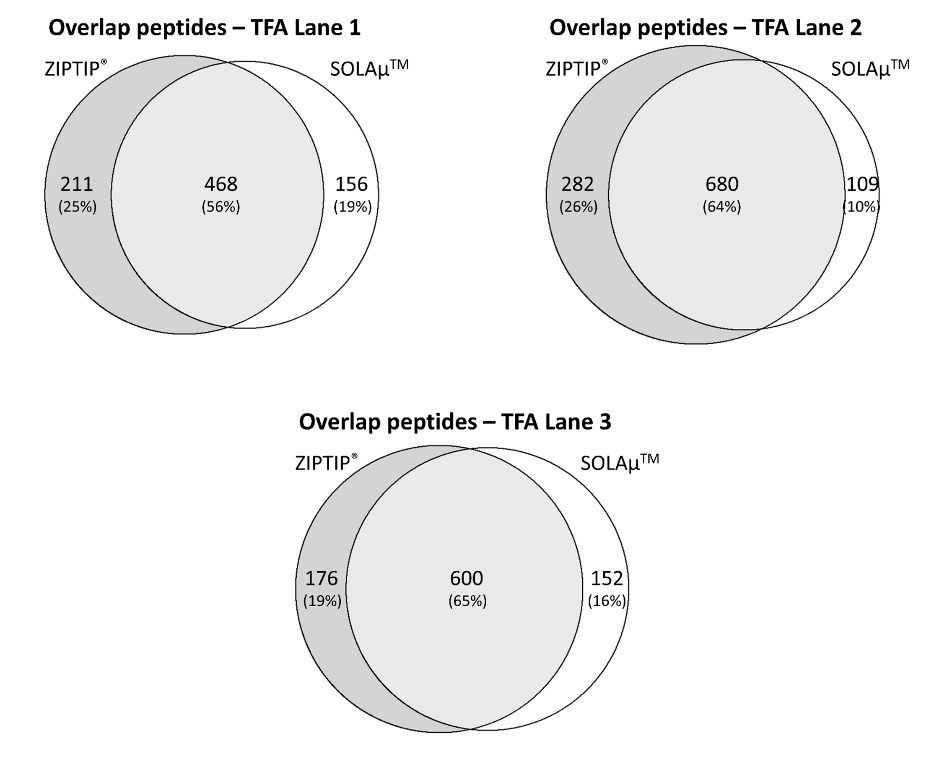


**Supplementary Fig. 2:** Venn diagram showing the overlap (percentage distribution) of identified peptides in the TFA fraction between six technical replicates. Three technical replicates were either purified by ZIPTIP^®^ pipette tips or SOLAµ^TM^ microtiter plates. On average 62±5 % of all identified peptides were detected with both SPE-based purification methods.


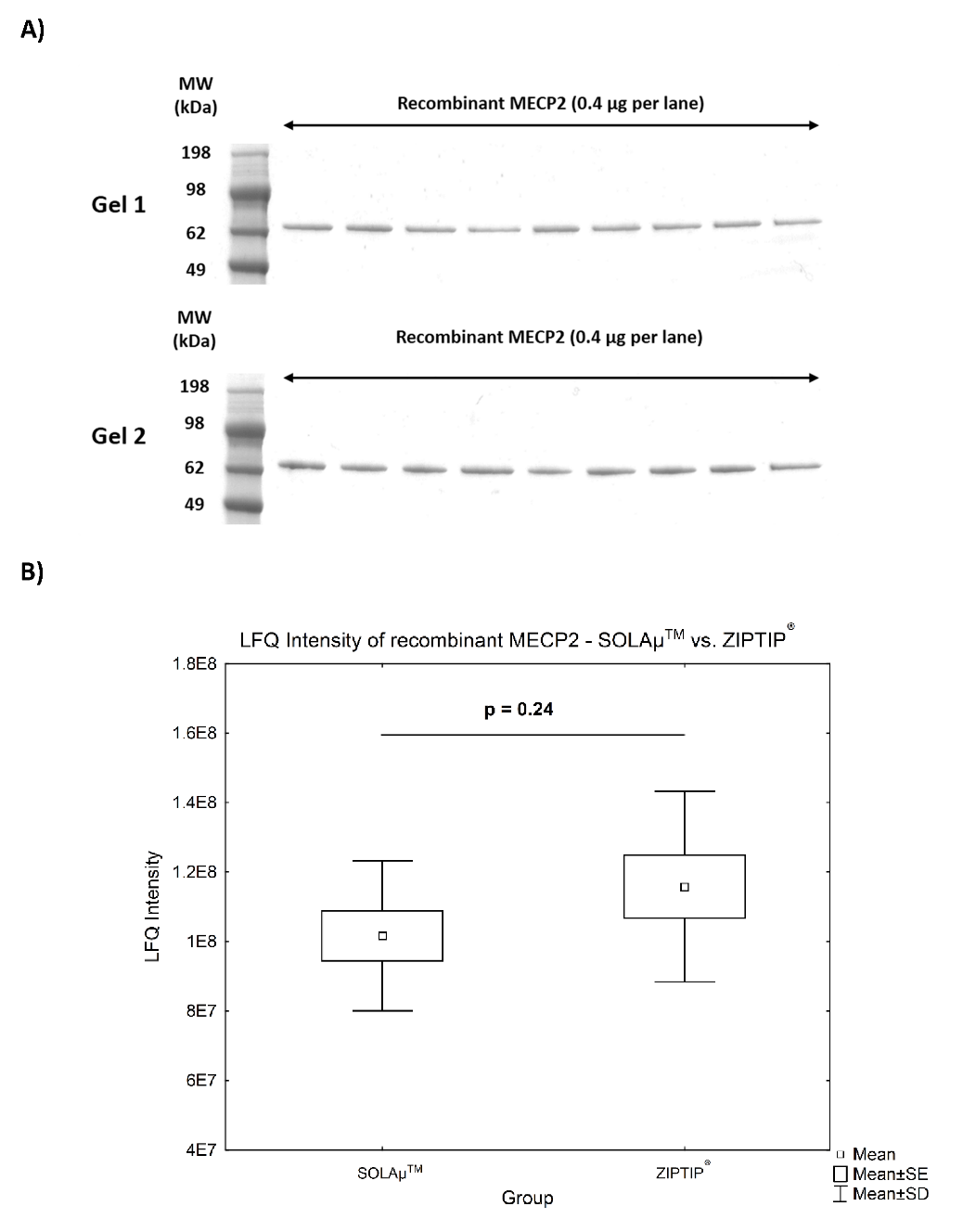


**Supplementary Fig. 3**: Tryptic in-gel digestion of recombinant protein *MECP2*. **(A)** 1D SDS page of recombinant protein *MECP2* (≈ 62 kDa) with 0.4 µg per lane (N=18). **(B)** Label-free quantification (LFQ) result of recombinant protein *MECP2* after peptide enrichment by either ZIPTIP^®^ pipette tips (N=9) or SOLAµ^TM^ microtiter plates (N=9). LFQ analysis showed on average a slightly higher abundance in ZIPTIP^®^-purified replicates, but no significant difference (p = 0.24; N=9 per group) was found between both SPE-based purification methods.
