# Supplementary material for: Comparison of Two Solid-Phase Extraction (SPE) Methods for the Identification and Quantification of Porcine Retinal Protein Markers by LC-MS/MS
Source: Int J Mol Sci. 2018 Dec 3;19(12):3847. doi: 10.3390/ijms19123847 (PMC6321002; doi:10.3390/ijms19123847)
Supplement: Supplementary file 1 [file ijms-19-03847-s001.zip › supplementary files/Supplementary Tab. 1.docx]

**Supplementary Tab. 1:** LFQ intensities and p-values of the 25 glaucoma-associated marker proteins after purification and enrichment by either ZIPTIP^®^ C18 pipette tips or SOLAµ^TM^ microtiter plates.

|  | Proteins | Gene name | LFQ intensity - SOLAµ^TM^ | LFQ intensity - ZIPTIP^®^ | p-value |
| --- | --- | --- | --- | --- | --- |
| DDM | Reticulon-3 | RTN3 | 1,17*10^6^ ± 3,5*10^5^ | 1,23*10^6^ ± 2,8*10^5^ | 0,83 |
|  | Vesicle-associated membrane protein-associated protein B/C | VAPB | 5,78*10^5^ ± 2,7*10^5^ | 6,14*10^5^ ± 3,1*10^5^ | 0,89 |
|  | Guanine nucleotide-binding protein G(i) subunit alpha-2 | GNAI2 | 1,12*10^7^ ± 1,9*10^6^ | 7,75*10^6^ ± 2,8*10^6^ | 0,15 |
|  | ATP-dependent 6-phosphofructokinase, muscle type | PFKM | 5,16*10^6^ ± 1,0*10^6^ | 6,26*10^6^ ± 1,0*10^6^ | 0,26 |
|  | Retinol-binding protein 3 | RBP3 | 1,32*10^7^ ± 1,9*10^6^ | 1,17*10^7^ ± 3,2*10^6^ | 0,52 |
|  | Pyruvate dehydrogenase E1 component subunit beta, mitochondrial | PDHB | 8,44*10^5^ ± 3,5*10^5^ | 6,11*10^5^ ± 8,0*10^4^ | 0,32 |
|  | ADP/ATP translocase 3 | SLC25A6 | 2,34*10^7^ ± 1,0*10^7^ | 1,89*10^7^ ± 7,8*10^6^ | 0,58 |
|  | Endoplasmin | HSP90B1 | 7,62*10^6^ ± 7,1*10^5^ | 7,23*10^6^ ± 7,3*10^5^ | 0,54 |
|  | Rab GDP dissociation inhibitor alpha | GDI1 | 7,84*10^6^ ± 1,4*10^6^ | 6,82*10^6^ ± 2,1*10^6^ | 0,52 |
|  | Myosin-11 | MYH11 | 3,09*10^6^ ± 1,6*10^5^ | 3,38*10^6^ ± 1,8*10^5^ | 0,11 |
|  | Vesicle-fusing ATPase | NSF | 1,64*10^6^ ± 1,2+10^6^ | 1,76*10^6^ ± 1,3*10^6^ | 0,91 |
|  | Small nuclear ribonucleoprotein Sm D3 | SNRPD3 | 8,28*10^6^ ± 1,8*10^6^ | 8,55*10^6^ ± 2,1*10^6^ | 0,87 |
|  | Heterogeneous nuclear ribonucleoprotein U | HNRNPU | 2,11*10^6^ ± 6,2*10^5^ | 2,74*10^6^ ± 1,2*10^6^ | 0,47 |
|  | Ras-related protein Rab-11B | RAB11B | 1,61*10^7^ ± 8,9*10^6^ | 1,01*10^7^ ± 4,8*10^6^ | 0,37 |
|  | Putative elongation factor 1-alpha-like 3 | EEF1A1P5 | 4,96*10^7^ ± 9,1*10^6^ | 4,79*10^7^ ± 9,3*10^6^ | 0,83 |
| TFA | PC4 and SFRS1-interacting protein | PSIP1 | 3,99*10^6^ ± 3,7*10^5^ | 3,75*10^6^ ± 6,6*10^5^ | 0,61 |
|  | Alpha-crystallin B chain | CRYAB | 2,74*10^7^ ± 3,2*10^6^ | 3,13*10^7^ ± 5,7*10^6^ | 0,36 |
|  | 60S acidic ribosomal protein P0 | RPLP0 | 3,01*10^5^ ± 3,9*10^4^ | 3,59*10^5^ ± 8,2*10^4^ | 0,34 |
|  | Histone H1.0 | H1F0 | 3,85*10^7^ ± 2,7*10^6^ | 4,13*10^7^ ± 2,5*10^6^ | 0,26 |
|  | Annexin A2 | ANXA2 | 2,34*10^6^ ± 1,7*10^5^ | 2,55*10^6^ ± 2,6*10^5^ | 0,54 |
|  | High mobility group protein HMG-I/HMG-Y | HMGA1 | 1,02*10^6^ ± 1,7*10^5^ | 1,17*10^6^ ± 1,2*10^5^ | 0,29 |
|  | Trifunctional enzyme subunit alpha, mitochondrial | HADHA | 1,30*10^6^ ± 6,8*10^4^ | 1,17*10^6^ ± 2,0*10^5^ | 0,33 |
|  | Methyl-CpG-binding protein 2 | MECP2 | 2,14*10^7^ ± 1,0*10^6^ | 2,43*10^7^ ± 8,2*10^5^ | 0,02 |
|  | 40S ribosomal protein S7 | RPS7 | 5,84*10^6^ ± 5,8*10^5^ | 5,53*10^6^ ± 3,4*10^5^ | 0,47 |
|  | High mobility group protein B1 | HMGB1 | 1,97*10^7^ ± 1,6*10^6^ | 2,41*10^7^ ± 3,8*10^6^ | 0,14 |
